# A Case of Cutaneous Diffuse Large B-cell Lymphoma

**DOI:** 10.4274/tjh.2013.0200

**Published:** 2014-06-10

**Authors:** Bülent Çetin, Ahmet Özet, Bülent Orhan, Tülay Tecimer

**Affiliations:** 1 Gazi University Faculty of Medicine, Department of Internal Medicine, Division of Medical Oncology, Ankara, Turkey; 2 Acıbadem University Faculty of Medicine, Department of Internal Medicine, Division of Medical Oncology, Bursa, Turkey; 3 Acıbadem University Faculty of Medicine, Department of Pathology, Bursa, Turkey

**Keywords:** non-Hodgkin lymphoma, B Cell neoplasms, Pharmacotherapeutics

## TO THE EDITOR

We report the clinical findings of a 55-year-old woman who presented with purple-reddish nodules on her face diagnosed as primary cutaneous diffuse large B-cell lymphoma (PCLBCL), which is very rarely seen in this area.

**Figure 1:**
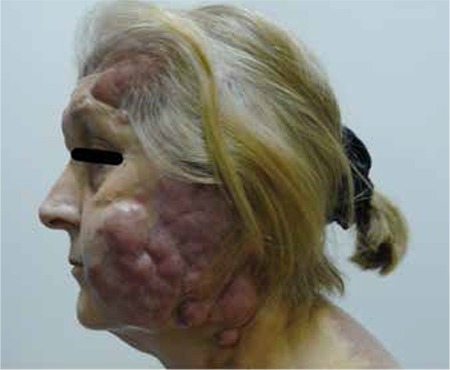
Patient with nodules and lumps of PCLBCL observed on the face and neck.

**Figure 2:**
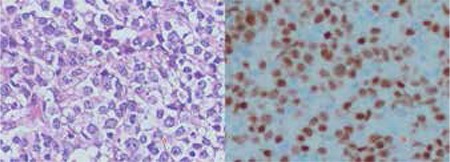
Neoplasm formed of large lymphoid cells developed in a diffuse pattern (H&E, 400x). B) Nuclear immunoreactivity by PAX5 in neoplastic cells (400x).

A 55-year-old woman applied to our oncology department after a brief episode of fever, general weakness, and erythematous patches and plaques in the facial area (Figure 1). Other associated symptoms and signs were general malaise and weight loss of 5 kg in 1 month. She had previously been well. A diagnostic biopsy showed an infiltration of large lymphoid cells staining positively for CD20, BCL-6, BCL-2, and PAX5, suggesting a diagnosis of diffuse large B-cell lymphoma (Figure 2). The cells were negative for CD5 and CD10. Renal and liver functions were normal. Bone marrow aspiration and biopsy showed no evidence of lymphoma. There was no other systemic organ involvement. The patient began receiving systemic chemotherapy with rituximab, cyclophosphamide, doxorubicin, vincristine, and prednisone. After 3 cycles of treatment, the large tumor showed no gradual improvement; this was suggestive of a lack of response. Rituximab plus ifosfamide, carboplatin, and etoposide chemotherapy (R-ICE) were therefore initiated. The patient was treated with 3 cycles of R-ICE chemotherapy. The patient declined additional treatment because her condition remained progressive despite the treatment, and she died soon after. Infomed consent was obtained.

PCLBCL is a rare primary cutaneous lymphoma characterized by a diffuse proliferation of large B cells consisting of centroblasts and immunoblasts, occurring most commonly on the legs [1]. PCLBCL affects elderly population with a female predominance. This type of lymphomas tend to develop on the lower limbs, predominantly as large dermal nodules or tumors, which are either solitary or multifocal and rapidly enlarging [1]. PCLBCL can also rarely occur at other cutaneous sites [2]. The prognosis of PCLBCL is poor, with a 5-year survival of 41%-58% [1,2,3,4]. The pathogenesis of PCLBCL is unknown. This tumor is composed of large monomorphic B cells that infiltrate the dermis. Tumor cells are usually strongly Bcl-2 positive [5], and Bcl-6 is also expressed in most cases with evidence of Bcl-6 gene mutations [5]. CD10 expression is only rarely detected in PCLBCL [5]. MUM-1 and FOX-P1 are invariably expressed by tumor cells in PCLBCL in contrast to primary cutaneous follicle center lymphoma [6]. PCLBCL often involves the leg area, while the face is unusual for presentation [4,7]. PCLBCL usually presents with a red or bluish-red tumor over the legs [3]. Clinically, patients present with cutaneous nodules, tumor-like lesions, or deeply infiltrated plaques [7]. Anthracycline-containing chemotherapy with rituximab should be considered for initial therapy. The incorporation of rituximab improves the response rates and overall survival [4,7].

In conclusion, we report an unusual clinical picture of PCLBCL on the face and trunk, a lymphoma that is generally localized on the legs.

## CONFLICT OF INTEREST STATEMENT

The authors of this paper have no conflicts of interest, including specific financial interests, relationships, and/ or affiliations relevant to the subject matter or materials included.
